# Distraction by unexpected sounds: comparing response repetition and response switching

**DOI:** 10.3389/fpsyg.2024.1451008

**Published:** 2024-10-02

**Authors:** Elena García-López, Fabrice B. R. Parmentier

**Affiliations:** ^1^Neuropsychology and Cognition Group, Research Institute of Health Sciences, Department of Psychology, University of the Balearic Islands, Palma, Spain; ^2^Balearic Islands Health Research Institute (IdISBa), Palma, Spain; ^3^School of Psychological Science, University of Western Australia, Perth, WA, Australia

**Keywords:** attention capture, auditory distraction, distraction, response, response repetition, response switching, oddball

## Abstract

Numerous studies using oddball tasks have shown that unexpected sounds presented in a predictable or repeated sequence (deviant vs. standard sounds) capture attention and negatively impact ongoing behavioral performance. Here, we examine an aspect of this effect that has gone relatively unnoticed: the impact of deviant sounds is stronger for response repetitions than for response switches. Our approach was two-fold. First, we carried out a simulation to estimate the likelihood that stimuli sequences used in past work may not have used balanced proportions of response repetition and switch trials. More specifically, we sought to determine whether the larger distraction effect for response repetitions may have reflected a rarer, and thereby more surprising, occurrence of such trials. To do so, we simulated 10,000 stimuli sets for a 2-AFC task with a proportion of deviant trial of 0.1 or 0.16. Second, we carried out a 2-AFC oddball task in which participants judged the duration of a tone (short vs. long). We carefully controlled the sequence of stimuli to ensure to balance the proportions of response repetitions and response switches across the standard and deviant conditions. The results of the stimuli simulation showed that, contrary to our concerns, response switches were more likely than response repetitions when left uncontrolled for. This suggests that the larger distraction found for response repetition in past work may in fact have been underestimated. In the tone duration judgment task, the results showed a large impact of the response type on distraction as measured by response times: Deviants sounds significantly delayed response repetitions but notably accelerated switches. These findings suggest that deviant sound hinder response repetition and encourage or bias the cognitive system towards a change of responses. We discuss these findings in relation to the adaptive nature of the involuntary detection of unexpected stimuli and in relation to the notion of partial repetition costs. We argue that results are in line with the binding account as well as with the signaling theory.

## 1 Introduction

Our environment is filled with a multitude of sensory stimuli that constantly compete for our attention. Efficient functioning often requires maintaining our attention focused on a task while filtering out task-irrelevant stimuli (e.g., ignoring the background noise in a busy place while reading a book). However, the complete blocking of extraneous stimuli would not be adaptive, for such stimuli can provide signals calling for a change in our actions. For example, a rodent foraging for food will, upon the occurrence of a sudden and unexpected sound, interrupt its behavior and orient its attention towards the source of this sound (e.g., Sokolov, 1963). Abundant research effort has been dedicated to both attentional functions: selective attention (Treisman, 1964; Driver, 2001) and change detection (Escera et al., 1998; Corbetta and Shulman, 2002; Näätänen et al., 2007; Näätänen, 2018). While their equilibrium offers adaptive advantages, it can come at a price when an unexpected stimulus of no relevance captures our attention away from the task at hand and causes a decrement in task performance (Parmentier, 2016). This decrement typically takes the form of a lengthening of response times (and sometimes a reduction in response accuracy) and is the consequence of multiple underpinning mechanisms (sensory, attentional and motor). In this study, we refer to the impact of the deviant sounds on performance as distraction. The aim of this study was to further our understanding of the impact of deviant sounds by focusing on a rarely addressed, but relevant, issue: the impact of unexpected sounds on our behavior as a function of whether the task requires the repetition of a behavior versus its modification.

In cognitive research, the impact of deviance stimuli is typically studied using oddball tasks in which participants categorize target stimuli while instructed to ignore task-irrelevant information. The task can take several forms. In the cross-modal version, a task-irrelevant stimulus (e.g., a sound) shortly precedes a target stimulus presented in a distinct modality (Escera et al., 1998; Ljungberg and Parmentier, 2012; Parmentier and Gallego, 2020; Weise et al., 2023). Participants are asked to categorize the visual target (e.g., a digit to be categorized as odd or even). In the unimodal version, the task may consist of a stimulus that conveys both task-relevant and task-irrelevant information (for example, participants may be required to categorize the duration of sounds as short or long while ignoring other aspects such as their pitch; Schröger, 1996; Roeber et al., 2003; Berti, 2008; Volosin and Horváth, 2020). A key feature of the oddball paradigm is that the same task-irrelevant stimulus or feature is used in a majority of trials (standard condition), while it deviates from this stimulus on rare and unexpected occasions (deviant condition). A plethora of studies show that, relative to standard stimuli, deviant stimuli trigger specific electrophysiological responses (Schröger, 1996; Escera et al., 1998; Schröger and Wolff, 1998b; Schröger and Wolff, 1998a; Berti and Schröger, 2001) and the lengthening of response times in the primary task (Schröger, 1996; Escera et al., 1998; Parmentier, 2014). Research suggests that these effects originate from the violation of sensory predictions (Bendixen et al., 2008; Bubic et al., 2009; Parmentier et al., 2011; Schröger et al., 2015b; Schröger et al., 2015a), which trigger a rapid but transient inhibition of motor actions (Wessel, 2017; Dutra et al., 2018; Finzi et al., 2018; Vasilev et al., 2019) reminiscent of the circuit breaker concept (Corbetta and Shulman, 2002; Corbetta et al., 2008; Verbruggen et al., 2014), the orienting to (and reorientation from) the unexpected stimulus (Parmentier, 2014), and that the impact of deviant sounds appears unaffected by response predictability (Parmentier and Gallego, 2020).

While a large proportion of past research using the oddball task focused on brain responses to stimulus deviance (as briefly highlighted in the previous paragraph), our study focuses on the behavioral manifestation of distraction. We see this line of work as complementary to electrophysiological studies and relevant for at least three reasons. First, to cognitive psychologists interested in the manifestation of cognitive mechanisms in performance, it is crucial to measure behavior and achieve an understanding of the factors modulating it. Second, evidence shows that the effect of deviant stimuli on behavioral performance is not a simple byproduct of their electrophysiological effects. For example, behavioral performance is sensitive to certain manipulations in the absence of any key electrophysiological variation (e.g., Wetzel et al., 2013) while variations in electrophysiological responses have been reported in the absence of behavioral effects (e.g., Getzmann et al., 2013). Finally, behavioral effects, because they are measured from the last stage of processing (the execution of a response), can manifest a mixture of effects (including effects occurring later than, or not correlated with, electrophysiological responses such as MMN, P3a or RON) and lend themselves to theoretical explanation that combine multiple factors (e.g., Parmentier, 2016; Parmentier et al., 2018).

One key aspect of the oddball tasks described above is the requirement for participants to produce motor responses to target stimuli (almost invariably binary responses). This is of interest given the adaptive character often attributed to the involuntary orienting response triggered by the occurrence of an unexpected stimulus (Sokolov, 1963; Sokolov, 2001). Recent advances have put forward convincing evidence that expected sounds trigger a very fast and temporary inhibition of motor actions (Wessel and Aron, 2013; Wessel and Aron, 2017; Dutra et al., 2018). Recent evidence suggests that unexpected sounds induce both global inhibition of motor responses and an orienting response (Wessel and Aron, 2013; Wessel and Aron, 2017; Dutra et al., 2018; Wessel, 2018a). For instance, studies using TMS stimulation have shown a reduction in motor-evoked potentials (MEPs) approximately 150 ms after an unexpected sound presentation (Wessel and Aron, 2013; Dutra et al., 2018; Iacullo et al., 2020). Consistent evidence from studies measuring eye movements in reading and scanning tasks shows an increase in fixation duration following the presentation of a deviant sound (Vasilev et al., 2019; Vasilev et al., 2021; Vasilev et al., 2023).

The recent developments described above highlight the relevance of considering motor actions when ascertaining the impact of unexpected sounds on behavioral performance. Interestingly, some earlier evidence, somewhat overlooked, reported an interesting and theoretically important observation: Deviance distraction appears to be greater when participants repeat the response produced on the previous trial compared to when they switch responses. Reanalyzing the data from previous experiments in which participants categorized the duration of a sound (short vs. long) while ignoring rare and unpredictable changes in pitch, Roeber et al. (2005) reported a larger deviance distraction effect for response repetitions than for response changes. The authors interpreted this finding as an indication of a response bias towards change: When a change occurs in the stimuli, the cognitive system leans towards changing its response. If the appropriate action is to repeat the previous response, this bias must be counteracted, and the response is thereby delayed. This interpretation certainly seems appealing and adaptive, for a change in one’s immediate surroundings may result in one’s ongoing behavior being no longer optimum or adequate. It is worth noting that this notion fits with the general observation that the cognitive system extracts contingencies from the environment to economize resources and facilitate the preparation of motor responses. Indeed, it is typically observed that response times decrease when a previous stimulus–response is repeated (Bertelson, 1963; Rabbitt and Vyas., 1973; Kleinsorge, 1999; Hübner et al., 2004; Roeber et al., 2005; Schuch and Koch, 2010; Koch et al., 2018). In the words of Kleinsorge (1999): “any change of a task feature that is part of the task representation subjects adopt will lead to a disruption of repetition based facilitation and tends to facilitate a response alternation” (p. 309). Hence, one may expect that deviant stimuli, by introducing a change from the previous trial, should disrupt response repetition while facilitating change, thereby resulting in the lengthening and shortening of response times, respectively.

However, it should be noted that Roeber et al.’s (2005) study was not designed for the specific purpose of addressing this question. Indeed, they revisited existing data sets from experiments in which the proportion of deviant trials was either set to 0.1 or 0.16, but in which the proportions of trials requiring response repetitions and switches were not quantified or controlled for. This means that the greater deviance distraction effect observed for response repetitions may, at least in part, reflect a lower probability of deviant trials requiring a response repetition. Under the assumption that response times may increase as the probability of a type of trial decreases (that is, as this type of trial yields greater surprise; Berti et al., 2004), it becomes important to ascertain whether the results of Roeber et al. (2005) may potentially be explained by an imbalance in the proportion of trial types in their study, and whether the effect can be replicated when trial sequences are tightly controlled.

The present study sought to address the issue highlighted above in two steps. First, to assess whether the statistical characteristics of the trial sequences used of Roeber et al. (2005) may have influenced the behavioral results they reported, we conducted two simulations in which we generated 10,000 sequences of trials under constraints like those of the authors’ study. In one, the proportion of deviant trials was set to 0.1, while it was 0.16 in to other (following the authors’ original description). Our aim was to obtain estimates of the likely relative probabilities of response repetitions and changes across standard and deviant trials. Of particular interest, we sought to compare the probability ratio between trials requiring response repetitions and response switches for deviant and standard trials. Our rationale was that if response repetitions were relatively more surprising in deviant trials compared to standard trials, then it is possible that past behavioral results may in part reflect this difference. Second, we carried out a new experiment to examine deviance distraction with respect to response repetition vs. change in a duration judgment experiment in which we controlled the sequences of trials to eliminate the possible bias of behavioral performance by differential levels of surprise by deviant trials in the response repetition vs. switch conditions.

## 2 Simulation

### 2.1 Methods

In each of our two simulations, we generated a total of 10,000 stimuli sets for a 2-alternative forced choice task (2-AFC), following criteria inspired from those of Roeber et al. (2005).

Each simulated stimuli set consisted of 1,400 trials. These simulations were run by using a quasi-random generation of stimuli responding to the rules described as follows. In each simulation, each set included 10% of the deviant trials (140 deviant trials), while in the other it included 16% (224 deviant trials). Consistent with Roeber et al.’s methodology, we controlled some key factors: Deviant trials were never presented on subsequent trials and the first three trials of each block of 280 trials were standard trials. In all sets, 50% of the trials corresponded to the long sound, while the remaining trials corresponded to the short sound. Additionally, to avoid variations in the concentration of deviant trials across different portions of the experiment, we ensured that two deviant trials were present in every successive group of 20 trials (which also included equal number of short and long sounds).

We calculated the ratio of proportions between trials requiring response repetitions and response switched for each sound condition (standard and deviant), to assess the level of surprise corresponding to response repetitions and switches within each sound condition. We hereafter refer to this measure as the repetition to switch ratio (R/S). To illustrate it, let us imagine that the probabilities of required repetitions and required switches were 0.04 and 0.06, respectively, in deviant trials, and were both 0.45 in standard trials. If so, then response repetition would be less surprising than response switching in deviant trials (0.06/0.04 = 1.5) than in standard trials (0.45/0.45 = 1). Our rationale is that the smaller the ratio of response repetition trials over response switch trials within a sound condition (standard or deviant), the more surprising the repetition (or vice versa, the greater the ratio, the more surprising the switch).

### 2.2 Results

**Figure 1:**
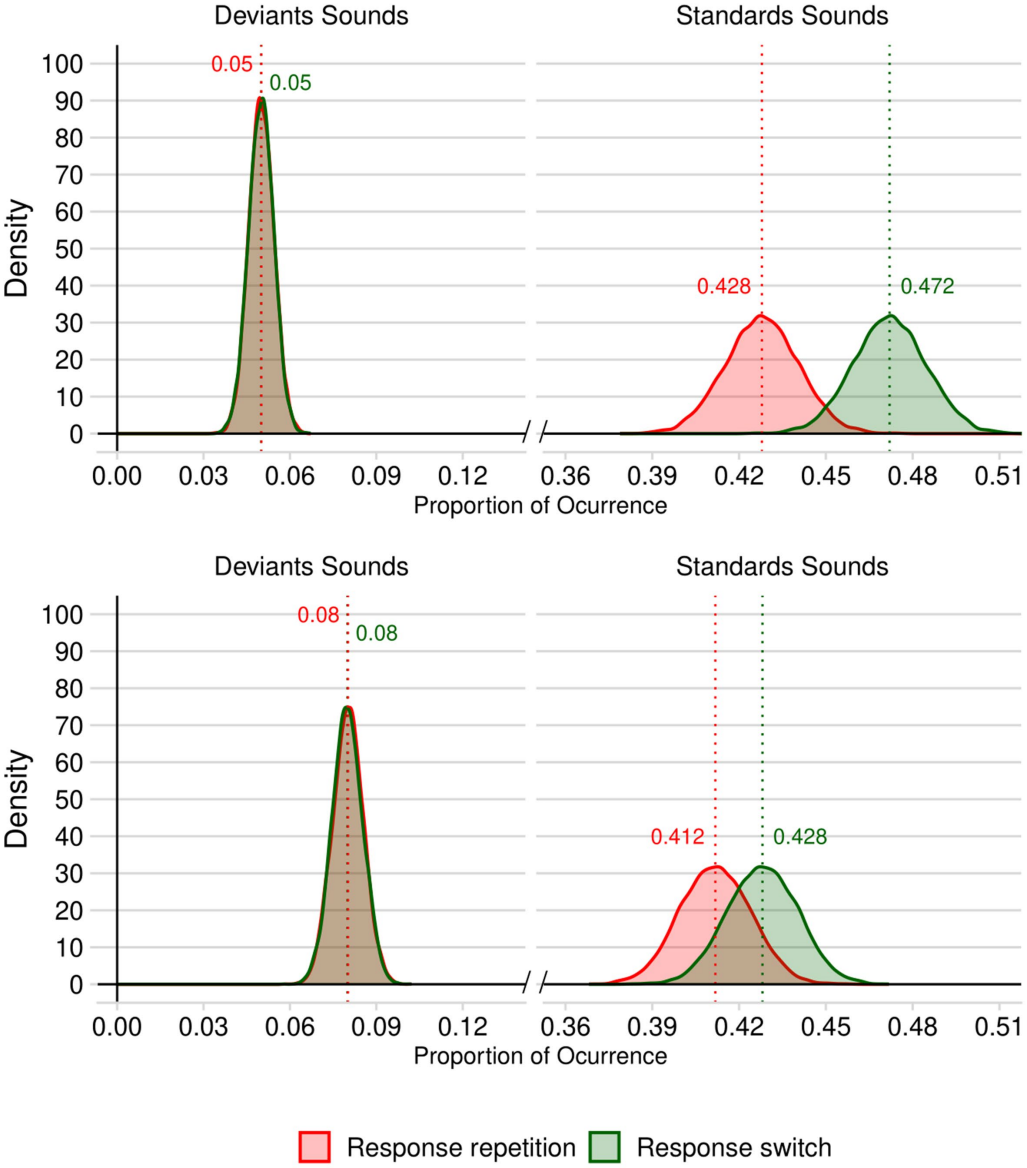
Density plots of the standard and deviant trials requiring response repetition and response switching in our simulated 10,000 stimuli sets with 10% of deviant trials (top panel) and with 16% of deviant trials (bottom panel). Numerical values appearing above each probability distribution represent the mean probability of each type of trial type.

In the 10% deviant trials simulation, we compared the R/S in the deviant and standard conditions using a two-tailed *t*-test for paired samples, which revealed a significant difference: *t*(9999) = 57.893, *p* < 0.001, *d* = 0.579 (95% CI: 0.558 to 0.600), *BF_10_* = ∞, with a greater R/S in the deviant condition (*M* = 1.017, *SD* = 0.180) than in the standard condition (*M* = 0.908, *SD* = 0.051). As visible from Figure 1 (top panel), these results reflects the fact that, while the proportions of deviant trials (repetition and switch) are comparable [*t*(9999) = 0.870, *p* < 0.385, *d* = 0.009 (95% CI: −0.011 to 0.028), *BF_10_* = 0.016], standard trials are more likely to require response switching than response repetition: *t*(9999) = 173.830, *p* < 0.001, *d* = 1.738 (95% CI: 1.707 to 1.769), *BF_10_* = ∞.

The results of the analysis of the 10,000 stimulus sets including 16% of deviant trials revealed results similar to those reported in the previous paragraph (see Figure 1, bottom panel). The R/S was significantly greater in the deviant condition (*M* = 1.011, *SD* = 0.135) than in the standard condition (*M* = 0.964, *SD* = 0.056), *t*(9999) = 32.327, *p* < 0.001, *d* = 0.323 (95% CI: 0.303 to 0.343), *BF_10_* = 6.630 × 10^213^. The proportions in each of the conditions are presented in Figure 1. While the proportions of deviant trials (repetition and change) were similar across response repetition and switch trials [*t*(9999) = 1.706, *p* < 0.088, *d* = 0.017 (95% CI: −0.0.003 to 0.037), *BF_10_* = 0.048.], standard trials are more likely to require response switching than response repetition: *t*(9999) = 66.539, *p* < 0.001, *d* = 0.017 (95% CI: 0.644 to 0.687), *BF_10_* = ∞.

In order to evaluate the likelihood that experiments using a 2-AFC task with a 10 and 16% of deviant trials would include more standard trials requiring response switches than response repetitions, we estimated the probability that a randomly selected value from the repetition response distribution would be greater than a randomly selected value from the response switching distribution. To do this, we employed a Monte Carlo simulation approach (Robert and Casella, 2004; Rubinstein and Kroese, 2016). This involved generating 100,000 random samples from each fitted normal distribution. To quantify the uncertainty of these estimates, we employed the bootstrap method (Efron and Tibshirani, 1994) with 10,000 resamples to calculate 95% confidence intervals. The results of this simulation revealed that the likelihood that standard trials required more response switches than response repetitions is 0.993 (95% CI: 0.992 to 0.993) when deviant trials represent 10% of all trials, and 0.827 (95% CI: 0.824 to 0.829) when they represent 16% of trials. Hence, relatively, we can be reasonably confident that the results of Roeber et al. (2005) were based on stimuli sets in which deviant trials requiring a response switch were more surprising than those requiring response repetition.

### 2.3 Discussion

Our findings indicate a likely imbalance in the proportion of trial types when generating sequences of characteristics similar to those described by Roeber et al. (2005) and of many other studies in the field. The analysis of the relative proportions or trials requiring a response repetition or a response switch in the deviant and standard conditions shows that repetitions are relatively less surprising than switches in deviant trials (and relatively more surprising in standard trials). Under the assumption that response times increase as the surprise yielded by a type of trial decreases (Jentzsch and Sommer, 2002; Mars et al., 2008), this finding may be relevant to interpret the results of Roeber et al. (2005). Contrary to what we initially envisaged, our simulation suggests that Roeber et al.’s (2005) findings are very unlikely to be explained by a greater degree of surprise yielded by deviant trials requiring response repetition. On the contrary, if anything, they suggest that the greater distraction effect for response repetition relative to response change reported by Roeber et al. (2005) may have been underestimated. Because an imbalance in trial types does appear to occur when generating the quasi-random sequences of typical 2-AFC oddball tasks, it is important to examine how deviance distraction may be shaped by response repetition and response change in the absence of variation in the proportions of such trials. To address this issue, we conducted a 2-AFC experiment in which participants categorized the duration of a tone while carefully controlling for the proportion of each trial type to ensure that deviant trials are not more surprising for one type of response (change) than for another (repetition). This approach should help mitigate the potential bias in behavioral performance resulting from different levels of surprise across condition in studies of this nature.

## 3 Experiment

In this experiment, participants judged whether target sounds were either long or short while ignoring rare and unexpected deviations in pitch (Roeber et al., 2005; Horváth et al., 2009; Berti et al., 2013; Getzmann et al., 2013; Leiva et al., 2015). In contrast to previous studies, we constructed the stimuli set to ensure comparable probabilities of response repetitions and response changes within each sound condition (standard & deviant), while setting the proportion of deviant trials to 0.1. We chose this proportion because our simulation suggests that it was the most likely to have affected performance in Roeber et al.’s (2005).

Based on the results of our simulation and Roeber et al.’s (2005) findings, we hypothesized that deviance distraction should be significantly greater for response repetitions than for response changes. Of special interest, we aimed to determine whether significant deviance distraction would be observed for response changes (as reported by Roeber et al., 2005) or whether such distraction would disappear when the proportion of trials requiring a response change is not inferior to that of trials requiring response repetition.

### 3.1 Methods

#### 3.1.1 Participants

A total of 46 healthy participants, six males (age, *M* = 20.5, *SD* = 2.86) and 40 females (age, *M* = 22.7, *SD* = 5.82) took part in this experiment. Forty-two were right-handed (four were left-handed). None of the participants reported auditory, neurological, or psychiatric impairments or conditions. All participants signed an informed consent form prior to taking part in the experiment. Their participation was rewarded with a financial compensation of 10 euros or course credit. The research was approved by the Ethics Committee of the University of the Balearic Islands (319CER23).

To ensure a sufficient sample size, we conducted an *a priori t*-test power analysis using GPower 3.1 (Faul et al., 2007). The power analysis was based on a Type I error probability of 0.05 and a power of 0.95. Given the abundant evidence of shorter RT for response repetition (as opposed to response change) in the literature, the hypothesis that deviant sounds facilitate response switching while hindering response repetition may reasonably be expected to generate an effect of medium effect size or greater. Under the hypothesis of a medium effect size (*d* = 0.5), the required sample size is of 45 participants. To bolster this estimate, we also computed the effect size of the difference in deviance distraction for response repetition and response change from the 42 young adults in the auditory duration judgment task of Leiva et al. (2015), which revealed *d* = 1.385. Hence, we would argue that our sample size (*N* = 46) was appropriate and conservative.

#### 3.1.2 Stimuli and procedure

All participants completed the oddball task in a sound-attenuated booth. The task was programmed using E-Prime 3.0 software (Psychology Software Tools, Pittsburgh, PA) (Psychology Software Tools, Inc., 2016). Sounds were generated as mp3 files (mono, 44,100 Hz, 32bit) and were presented diotically through headphones at an intensity of approximately 70 db SPL.

**Figure 2:**
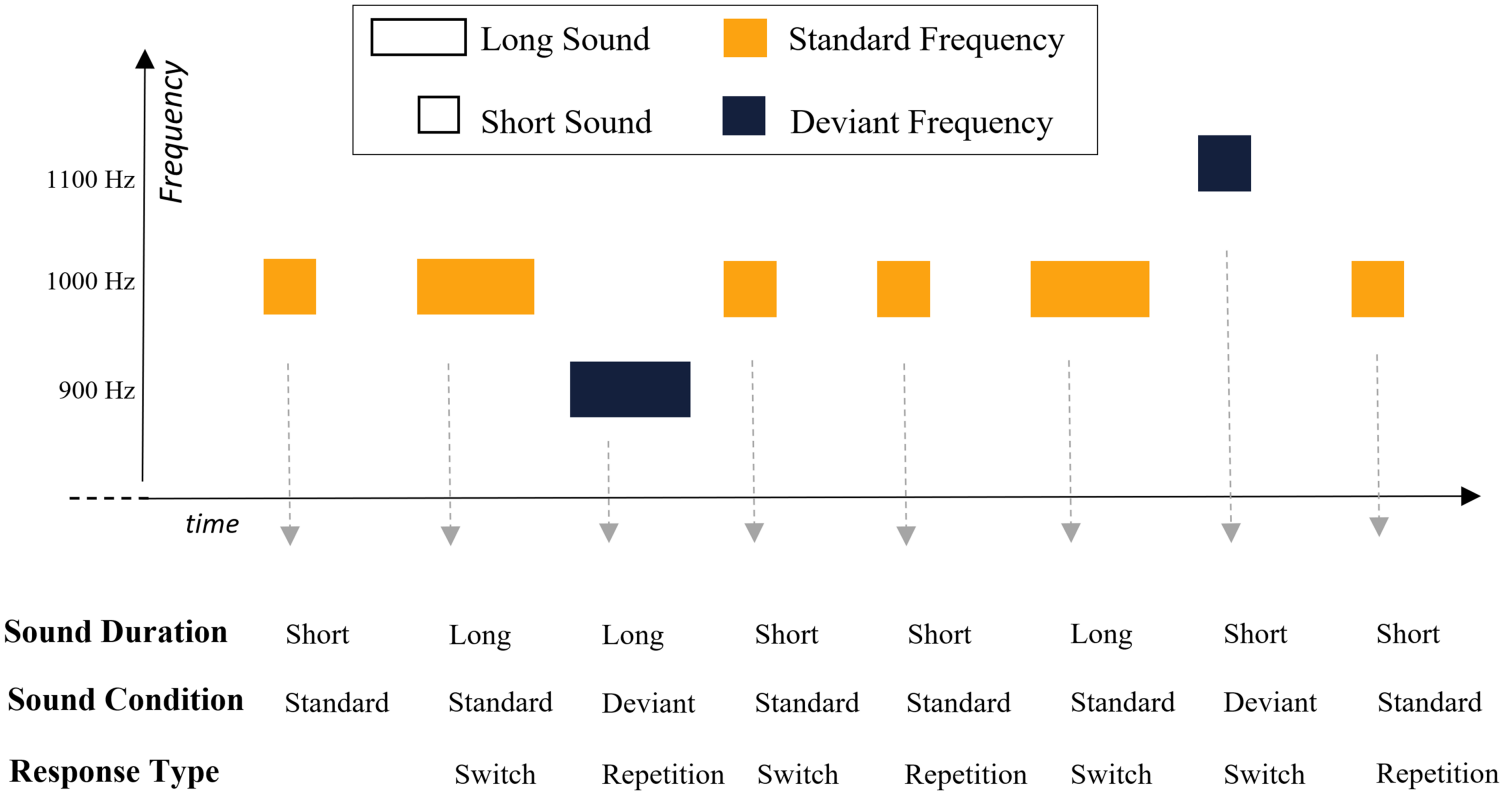
Schematic illustration of a sequence of trials in our 2-AFC task. Participants were asked to judge the duration of each sound (short vs. long) while ignoring rare and unexpected changes in auditory frequency. The lower part of the figure depicts the duration of each sound, the sound condition (standard vs. deviant) and whether the required response constituted a repletion of, or a switch from, the previous response.

In each trial, participants were asked to distinguish between short and long sounds (200 vs. 400 ms, respectively, equiprobable across the task). They were instructed to respond as quickly as possible while trying to make no error, using “X” and “M” keyboard keys (counterbalanced across participants). Three sounds were used. The standard sound, presented in 90% of trials, consisted of a 1,000 Hz sinewave tone. The deviant sound condition was made out two sinewave tones (900 Hz and 1,100 Hz), each presented in 5% of trials. All sounds were normalized and edited with 10 ms fade-in and fade-out ramps. The sounds frequency and duration condition were crossed orthogonally. Participants were required to respond to every sound based on its duration, irrespective of whether the sound was a standard or a deviant sound. A schematic illustration of an example of trials sequence is presented in Figure 2.

The crucial characteristic of the present study consisted in using carefully selecting sets of sequences that ensured comparable proportions of trials requiring response repetitions and response changes, in both standard and deviant conditions. We did this by selecting a subset from the 10,000 sequence sets generated for the simulation described earlier (hence the proportion of deviant trials was 0.1 across the task and within every successive group of 20 trials; short and long sounds were equiprobable and orthogonally crossed with the standard/deviant conditions). Importantly, we verified that no significant differences were observed between the proportions of occurrence of trials requiring response repetition versus response changes in our stimuli sets, both in the deviant condition [*t*(43) = 1.700, *p* = 0.096, *d* = 0.259 (95% CI: −0.046 to 0.562), *BF_10_* = 0.620] and in the standard condition [*t*(43) = 1.900, *p* = 0.064, *d* = 0.290 (95% CI: −0 0.017 to 0.593), *BF_10_* = 0.849].

The test phase consisted in 1400 trials divided in 5 blocks of 280 trials each. Participants were allowed to take a short pause between blocks if they wished to. In each trial, the target sound (200 or 400 ms long) was followed by a response window of 1,100 or 900 ms, respectively, (such that the inter-trial interval was always 1,300 ms). Responses were measured from the critical time at which short and long sounds could be distinguished (that is, 200 ms into the sound). Prior to the test phase, participants were presented with a practice phase consisting of a minimum of one block of 20 trials (18 standard trials, 2 deviant trials). The practice block was repeated until the participant responded correctly in at least 80%of the trials. The timing of the practice trials was as described above with the only difference that each trial was followed by a 1,000 ms screen on which a message indicated whether the response was correct (in green color), incorrect (in red color), or if no response (white color) had been detected. The screen background remained dark grey throughout the task.

### 3.2 Results

**Figure 3:**
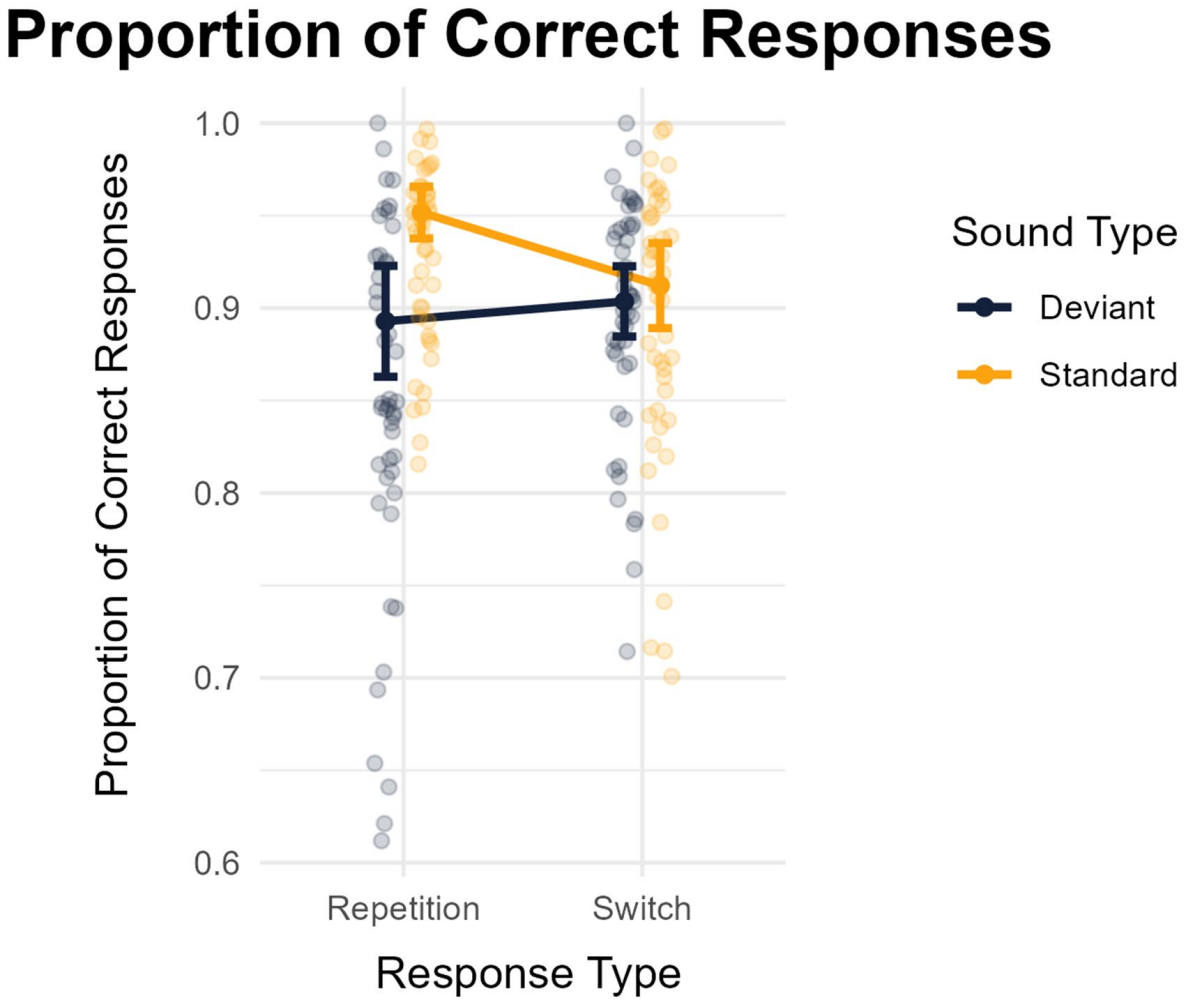
Mean proportions of correct responses as a function of the type of sound (standard or deviant) and whether participants repeated or changed their response relative to the previous trial. The error bars represent the 95% confidence interval of each mean.

The analysis of the proportion of correct responses showed a main effect of distraction (greater performance in the standard -*M* = 0.907, *SD* = 0.061- than in the deviant -*M* = 0.870, *SD* = 0.068- condition): *F*(1,45) = 27.940, *MSE* = 0.003, *p* < 0.001, *η^2^_p_* = 0.383, *BF*_*1*0_ = 1810.590. The main effect of response type was not significant: *F*(1,45) = 0.603, *MSE* = 0.002, *p* = 0.441, *η^2^_p_* = 0.013, *BF_10_* = 0.185 (*M* = 0.0892, *SD* = 0.070, for response switches, and *M* = 0.921, *SD* = 0.049, for response repetitions). Importantly, these two factors interacted significantly: *F*(1,45) = 23.415, *MSE* = 0.004 *p* < 0.001, *η^2^_p_* = 0.342, *BF_10_* = 65969.985. The analysis of this interaction revealed an absence of deviance distraction for response switches (standard, *M* = 0.892, *SD* = 0.075 vs. deviant, *M* = 0.895, *SD* = 0.064): *t*(45) = 0.305, *p* = 0.761, *d* = − 0.045 (95% CI: −0.334 to 0.244), *BF_10_* = 0.167; but marked deviance distraction for response repetitions (standard, *M* = 0.929, *SD* = 0.047 vs. deviant, *M* = 0.847, *SD* = 0.099): *t*(45) = 6.859, *p* < 0.001, *d* = 1.011 (95% CI: 0.651 to 1.364), *BF_10_* = 790584.677 (see Figure 3).

In analyzing response times (RTs), we employed ex-Gaussian analysis, which is regarded as an appropriate method to handle the positive skewness often observed in RT distributions (Heathcote et al., 1991; Spieler et al., 2000; Matzke and Wagenmakers, 2009). The ex-Gaussian model, which combines a normal distribution (characterized by parameters *μ* and *σ*) with an exponential distribution (characterized by parameter *τ*), provides a more nuanced characterization of RTs. Specifically, μ represents the central tendency of the normally distributed component, σ captures the variability of this component, and *τ* reflects the rate of occurrence of slower responses. The analysis was conducted using R Statistical Software (v4.3.1, R Core Team, 2023) using the MASS package (Venables and Ripley, 2002). For each subject and condition, we estimated the Ex-Gaussian parameters (μ, σ, and *τ*) using the Maximum Likelihood Estimation (MLE) method. The likelihood function for the Ex-Gaussian distribution was defined and optimized using the optim function in R, employing the Nelder–Mead method to minimize the negative log-likelihood of the model. Initial estimates for the parameters were provided based on the empirical characteristics of the data: the median response time for μ, the standard deviation for σ, and half the range of response times for τ.

**Figure 4:**
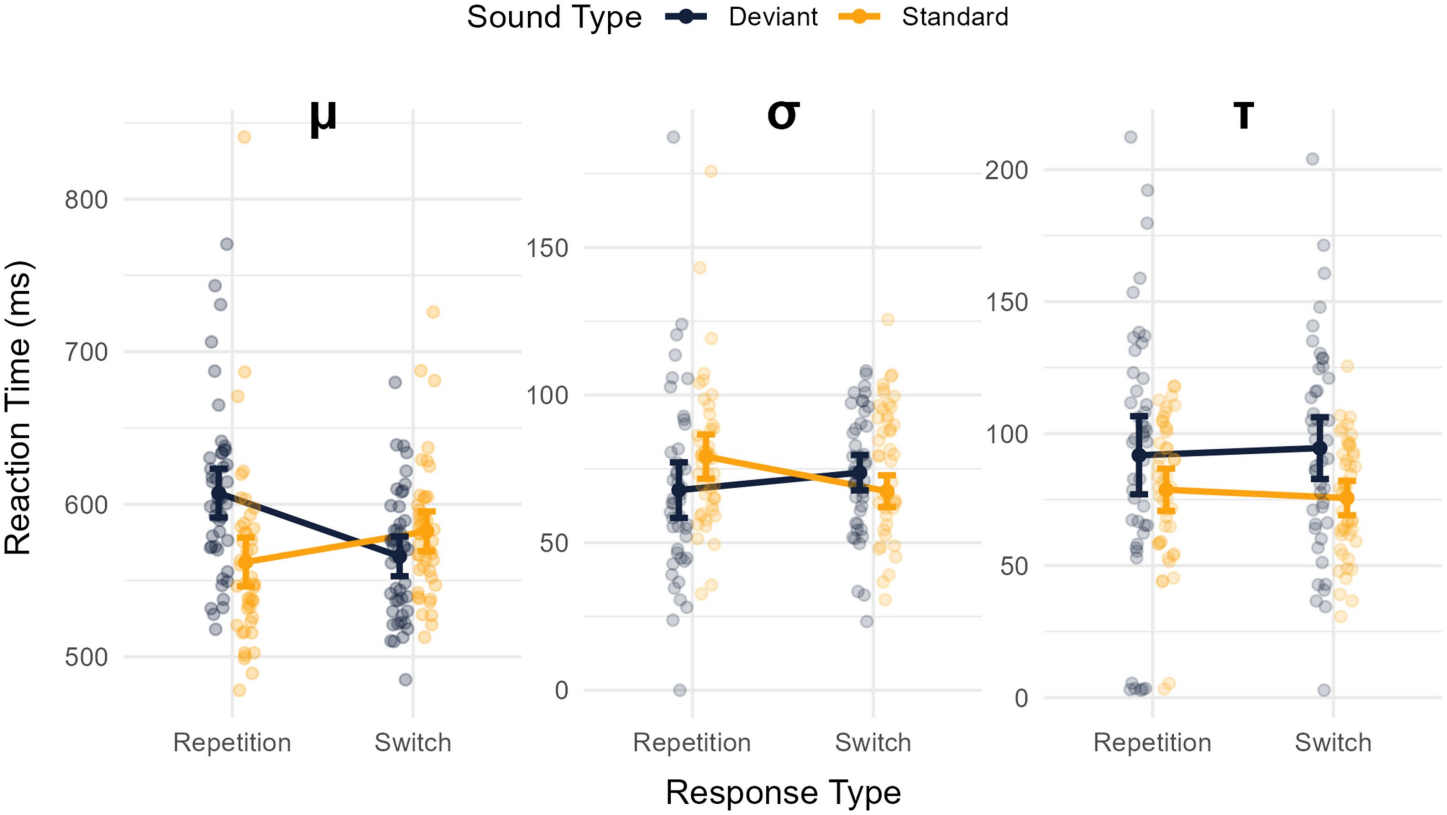
Mean values of the ex-Gaussian fitting parameters for response times (RT): *μ* (left panel), *σ* (middle panel), and *τ* (right panel). These variables are depicted as a function of the sound condition (standard vs. deviant) and the type of response (repetition vs. switch). Error bars represent the 95% confidence intervals for each mean.

The analysis of the μ revealed a main effect of sound type (longer RTs for deviant sounds) -*M* = 586.559, *SD* = 40.202- than for standard sounds -*M* = 572.202, *SD* = 49.898-: *F*(1,45) = 10.814, *MSE* = 876.797, *p* = 0.002, *η^2^_p_* = 0.194, *BF_10_* = 3.961. The main effect of response type was significant, with shorter RTs for response switches -*M* = 57.038, *SD* = 37.789- compared to response repetitions -*M* = 584.723, *SD* = 52.176-: *F*(1,45) = 4.868, *MSE* = 1078.813, *p* = 0.032, *η^2^_p_* = 0.098, though *BF_10_* = 0.904 was inconclusive. Importantly, the interaction between these factors was significant, *F*(1,45) = 41.689, *MSE* = 1047.806, *p* < 0.001, *η^2^_p_* = 0.481, *BF_10_* = 10508494.340 (see Figure 4, left panel). The analysis of this interaction revealed a greater *μ* in the deviant -M = 607.309, SD = 55.546- compared to the standard -M = 562.136, SD = 60.506- condition for response repetitions, *t*(45) = 6.284, *p* < 0.001, *d* = 0.927 (95% CI: 0.577 to 1.269), *BF_10_* = 123509.341], but significantly smaller μ in the deviant -M = 565.808, SD = 41.690- compared to standard -M = 582.267, SD = 43.060- condition for response switches, *t*(45) = −2.909, *p* = 0.006, *d* = −0.429 (95% CI: −0.729 to −0.125), *BF_10_* = 6.381.

The analysis of the *σ* revealed no main effects of sound type [*F*(1,45) = 0.788, *MSE* = 373.599, *p* = 0.379, *η^2^_p_* = 0.017, *BF_10_* = 0.219] or response type [*F*(1,45) = 0.815, *MSE* = 477.892, *p* = 0.371, *η^2^_p_* = 0.018, though *BF_10_* = 0.245]. However, the interaction between these factors was significant, *F*(1,45) = 11.874, *MSE* = 298.922, *p* < 0.001, *η^2^_p_* = 0.210, *BF_10_* = 13.698 (see Figure 4, middle panel). The analysis of this interaction revealed a smaller σ in the deviant condition -M = 67.790, SD = 31.761- compared to the standard -M = 79.141, SD = 25.307- condition for response repetitions, *t*(45) = 6.284, *p* < 0.001, *d* = 0.927 (95% CI: 0.577 to 1.269), *BF_10_* = 123509.341], but a greater σ in deviant -M = 73.701, SD = 20.135- compared to standard -M = 67.411, SD = 18.353- trials for response switches, *t*(45) = −2.909, *p* = 0.006, *d* = −0.429 (95% CI: −0.729 to −0.125), *BF_10_* = 6.381.

Finally, the analysis of *τ* revealed a main effect of sound type [*F*(1,45) = 10.439, *MSE* = 1126.641, *p* = 0.002, *η^2^_p_* = 0.188, *BF_10_* = 151.085] whereby τ was greater in the deviant -*M* = 186.279, *SD* = 78.215- than in the standard -*M* = 184.299, *SD* = 44.122- condition (see Figure 4, right panel). No main effect of response type was observed, *F*(1,45) = 0.003, *MSE* = 635.958, *p* = 0.954, *η^2^_p_* < 0.001, *BF_10_* = 0.156. No sound x response type interaction was found, *F*(1,45) = 0.707, *MSE* = 558.644, *p* = 0.405, *η^2^_p_* = 0.015, *BF_10_* = 0.250.

### 3.3 Discussion

The results of this experiment confirm that deviance distraction is greater in trials requiring the repetition of a response than a switch (as revealed by response times as well as the mean proportions of correct responses). One key aspect of our method was the use of stimuli sets that were designed to equate the proportions of trials requiring response repetitions and switches. Of interest, under these conditions, we observed significant deviance distraction for response repetitions but not for response switches. Of interest, the analysis of μ revealed that deviant sounds speeded responses switches relative to standard sounds. This result departs from the findings of Roeber et al. (2005) who found a reduced but nevertheless significant deviance distraction effect for response switches. The variability of RTs (σ) was smaller in the deviant condition compared to the standard condition for response repetitions, but the opposite pattern was observed for response switches. Taken together, these results indicate that conditions in which participants were slower also exhibited greater consistency. Finally, the tail of the RT distribution (τ) was revealed to be longer in the deviant condition compared to the standard condition, irrespective of the type of response. We discuss these findings and their implications in the next section.

## 4 General discussion

In this study, we sought to explore whether the behavioral distraction yielded by auditory deviance in a tone duration judgment task is modulated by response characteristics. More specifically, we compared deviance distraction as a function of whether participants repeated their previous response or switched responses. Our work followed the footsteps of Roeber et al. (2005) who were the first to point out comparatively greater RT differences between deviant and standard conditions for response repetitions than for response switches. One outstanding issue, however, concerned the statistical properties of the sequences used in prior work. Indeed, prior work analyzed performance based on the type of response (repetition vs. change) in *a posteriori* fashion, hence the proportion of trials requiring the two types of response was not reported or controlled for. It is therefore possible that Roeber et al.’s findings may have been, at least in part, modulated by the use of distinct relative proportions of response repetitions and changes within the two sound conditions (deviant & standard). For example, one may argue that if response repetitions are less frequent than response changes, deviant trials requiring a response repetition would constitute the most surprising type of trial in the task and therefore results in longer RTs. Alternatively, if response changes are the least frequent scenario, then the greater deviance distraction observed for response repetitions may in fact have been underestimated. To determine how likely response repetition and change trials are in a typical oddball task, we generated a large set of sequences based on commonly used constraints in the field (2-AFC tasks with a low proportion of deviant trials, no consecutive deviant trials, and equal proportions of short and long sounds). The density curves produced revealed that response repetitions are, in fact, less likely than response changes in a 2-AFC oddball task if generated quasi-randomly. The results of Roeber et al. (2005) could therefore hardly be explained by the likely statistical characteristics of the sequences they used. If anything, the striking modulation of deviance distraction by the type of response may have been underestimated in their study.

The results of our experiment expand the findings of Roeber et al. (2005) by showing a larger deviance distraction effect for response repetitions when sequences are carefully controlled to equate the proportions of trials requiring response repetition and switch. Interestingly, while Roeber et al. (2005) reported significant deviance distraction for both response repetitions and response switches (but comparatively less in the latter) for response times, we found strong deviance distraction for response repetitions but the opposite effect for response switches. That is, deviant sounds speeded up responses switches relative to the standard condition. This finding is compatible with the idea that the modulation of distraction by the type of response may have been underestimated in Roeber et al.’s (2005) study. From a methodological perspective, the key finding here is that the greater impact of deviant sounds on RTs for response repetitions reported by Roeber et al., cannot be explained by a relatively less frequent occurrence of trials requiring response repetitions than response changes.

The analysis of the ex-Gaussian parameters of the response times distribution revealed an interesting pattern. While *μ* and *σ* exhibited significant sound x response type interactions, *τ* was only sensitive to the main effect of the sound condition. This suggests that the central tendency measure of the RT distribution and RT variability are determined by cognitive mechanisms involved in the interplay between auditory stimuli and the planning/production of responses. In contrast, τ is only affected by auditory deviance, which suggests that it is not sensitive to the same variables or their interaction. The mapping of ex-Gaussian parameters onto specific cognitive functions is somewhat hazardous. Some authors have argued that ex-Gaussian fitting should be used as a descriptive tool rather than a theoretical model (Matzke and Wagenmakers, 2009; Rieger and Miller, 2020). There is to our knowledge no general agreement on the cognitive underpinnings of τ, and interpretations are multiple. For example, in response-compatibility studies, some have interpreted it as the sign of lapses of attention (e.g., Leth-Steensen et al., 2000), others as impairments of cognitive energy regulation (Sergeant, 2005), and others as an impaired speed of information accumulation for response production (Matzke and Wagenmakers, 2009). Whatever cognitive mechanisms may be responsible, the different pattern of results observed for μ and σ on the one hand, and τ on the other, does support the notion of an effect of auditory deviance independent of the mechanisms involved in the response production. While the behavioral manifestation of deviance distraction as measured by μ was modulated by the type of response (repetition/switch), this should not be taken as a direct measure of the degree to which unexpected sounds capture attention in the first place. Instead, it should be regarded as a moderating factor that manifests itself at the end of the processing chain, namely, the response stage. There is solid evidence suggesting that auditory deviance triggers a fast and transient general inhibition of motor activities (Wessel and Aron, 2013; Wessel, 2018b; Wessel, 2018a; Vasilev et al., 2021; Vasilev et al., 2023) shortly followed by an involuntary shift of attention towards the unexpected stimulus and a subsequent reorientation of attention towards the task at hand (Schröger, 1996; Parmentier, 2014). That we observed no distraction effect for μ in the response switch condition (in fact, we found facilitation) does not imply that these mechanisms were not at play. Response times are, by definition, a measure taken at time *t* that can capture the combined manifestation of multiple effects, some potentially of opposite polarity. Of relevance, Roeber et al.’s (2005) found deviant-induced MMN, P3a and RON for both response switch and repetition trials, suggesting the action of mechanisms that are not response-dependent. Intriguingly, they also found that the P3a amplitude was larger, and the RON delayed, in the response switch compared to the repetition response trials. Because the P3a response occurred about 100 ms after the key target information was delivered (i.e., the point at which the sound either stopped or continued, determining whether it was short or long), it is possible that some early response-related mechanism was at play that subsequently affected RON. A full discussion of these EEG results would fall outside the scope of our study. Altogether, the evidence does suggest that deviant sounds bias the cognitive system toward a change of response, thereby facilitating response switching and hindering response repetition, and that this impact can be of an amplitude sufficient to overcome the cost of earlier processes that contribute to lengthening response times.

One particularly relevant question relates to the theoretical meaning of the impact of deviant stimuli on response repetition/switching. One may argue that deviance, by introducing a change relative to recent past events, biases the cognitive system toward a change of behavior (thereby making behavioral repetition more difficult). There certainly is evidence suggesting that a change in stimuli induces a change in responses while the cognitive system will favor repeating a behavior when faced with the same stimulus (Kleinsorge, 1999; Quinlan, 1999). One may also argue that if one’s surrounding environment remains constant, the repetition of one’s behavior may be facilitated because it most likely constitutes an appropriate course of action. However, in the face of an unexpected change, it is adaptive to interrupt ongoing actions. In the words of Wessel (2017): “Evolutionarily, it makes sense for the cognitive system to implement rapid interruption of the ongoing task set in case of an expectancy violation” (p. 13). In sum, stimulus deviance may facilitate a change of response, hinder response repetition, or both. The contention that deviance may facilitate a change in behavior is not unprecedented in the oddball task, as it can also be observed when catch trials are introduced in the oddball task. Indeed, Parmentier (2016) demonstrated that in oddball tasks in which half the trials involve no target stimulus and participants must therefore withhold the production of a response, deviant stimuli produced longer RTs than standard stimuli in post-Go trials but produced the opposite effect in post No-Go trials. Here too, deviant sounds appear to help disengaging from the response mode at play on the previous trial. Deviant sounds help disconnect from the response inhibition mode at play on a No-Go trial, thereby facilitating the production of a response on the subsequent Go trial. In contrast, a deviant sound presented after a Go trial appears to hinder the perpetuation of the action mode. In sum, deviant sounds, by virtue of introducing an unexpected change in the auditory context, appear to make harder the repetition of one’s actions while facilitating the execution of a different action. Put differently, responses in our task were fastest when both sounds and responses were repeated from one trial to the next or when both changed. In contrast, responses were comparatively slower if one of the two elements, sound or response, changed. Our results therefore bare a direct functional similarity with several studies examining the partial repetition cost (Hommel, 1998; Hommel and Colzato, 2004; Hommel, 2007; Huffman et al., 2020; Weissman et al., 2023). In these studies, each trial consists in the presentation of a cue prompting participants to prepare a specific response (left or right key press). A stimulus presenting specific feature (e.g., color) to which participants must respond by executing the prepared response (Weissman et al., 2023). A second stimulus is then presented to which participants must now respond based on a pre-established stimulus–response mapping (e.g., if the stimulus is blue, press the left key). Findings from such studies show that the repetition of both stimulus color and response, or the change of both color and response, yield the fastest responses, while responses to partial repetitions (repeated color but change of response, or change of color but repeated response) are slower. Though these tasks differ from ours in many respects, the similarity is striking and opens interesting theoretical considerations, which we discuss below.

We can see two, non-mutually exclusive, theoretical frameworks capable of accounting for our results. The first is the binding or event-file theory (Hommel, 1998; Hommel, 2004; Hommel, 2022). According to this theory, the cognitive system integrates automatically task features (task-relevant or not), such as stimulus features and the response, into a bound representation or event file. Repeating a stimulus on the next trial triggers the automatic retrieval and activation of the associated response. If the required response matches the response automatically retrieved, the response latency will be short. However, if the required response is the alternative response to that activated, response latency will be longer because top-down control will be required to solve the conflict (Frings et al., 2020), and/or because unbinding is required to separate the repeated feature from the response (Hommel, 2004). Finally, if both stimulus and response change from one trial to the next, the theory posits a fast response because the creation of a new binding is rapid and efficient. A plausible alternative to the latter proposition may be offered based on the notion of lateral inhibition at the level of response representations (Botvinick et al., 2001; Usher and McClelland, 2001). The latter would predict that, in tasks involving two mutually exclusive responses, an inhibitory link between the two may form, with the activation of one response rapidly suppressing the other. Similarly, in the case of a conflict, the top-down inhibition of one would thereby result in the activation of the other.

A second framework may be relevant to account for our results: the signaling theory (Fletcher and Rabbitt, 1978; Usher and McClelland, 2001; Notebaert and Soetens, 2003; Huffman et al., 2020). According to this theory, partial repetition costs reflect a decision-making heuristic whereby the cognitive system codes each new stimulus as a repetition or an alternation relative to the previous stimulus. Hence, in a 2-AFC task, a stimulus repetition would automatically result in the selection of the same response, whereas a change in stimulus would elicit the activation of the alternative response. While this account and the binding account yield the same predictions in 2-AFC tasks, evidence indicates that they may both contribute to the partial repetition cost (Weissman et al., 2023).

Both accounts can be translated to our task. Let us illustrate it with an example. According to the binding account, upon the presentation of the standard sound of a short duration (to which participants would for example respond by pressing the X key), a memory trace of the associate link between this sound and the response is created. If the sound is repeated on the next trial, it would automatically potentiate the repetition of the same response (X), thereby reducing response latency. In contrast, if a deviant sound of short duration (change in stimulus but requiring response repetition), or a standard sound of long duration (stimulus repetition but change of response) is presented, the recent binding activated by the repeated feature is no longer helpful and introduces a conflict that delays the response. Finally, a deviant sound of long duration would yield no conflict because neither element (stimulus nor response) matches the previous binding. In this situation, the creation of a new binding is fast, and response latency would be short. According to the signaling account, upon the repetition of the standard sound, a heuristic is applied that biases the response selection towards the same response as on the previous trial. If that response matches the appropriate response, response latency is short. However, if it does not, cognitive control would be applied to suppress the automatically activated response and select the alternative response. In contrast, a deviant sound would be coded as an alternation of stimulus and the heuristic would therefore bias the response selection towards the alternative response to that produced on the previous trial. If this bias leads to the appropriate response, the response latency will be short. However, if the bias conflicts with the appropriate response, suppression of the irrelevant response and selection of the relevant one will first have to be carried out before a response is produced, thereby leading to a longer response latency.

In sum, the present study (1) replicates Roeber et al.’s (2005) interesting finding by confirming that deviance distraction is modulated by the type of response; and (2) that this differential effect is greater still when using stimuli sets in which trials requiring response repetitions and switches are equiprobable (to the extent that deviant sounds speed up responses in response switch trials). The precise control of the probabilities of response repetitions and switches (while controlling for the equiprobable presentation of the short and long stimuli and specific proportions of standard and deviant trials) is especially difficult to achieve in a 2-AFC oddball task, for response repetitions trials are naturally more frequent than response switch trials in the standard condition. From a methodological perspective, the key finding of this study is that differences in these probabilities cannot account for the differential degree of deviance distraction on response repetitions and response switches reported by Roeber et al.’s (2005). Hence, while we show that controlling for these probabilities appears to enhance the effect, such a level of control is not mandatory to observe it. Finally, our study offers some avenues for future research. Specifically, it may be theoretically interesting to seek to examine further the potential distinction between the mechanisms facilitating response change from those hindering repetition (something the typical 2-AFC oddball tasks does not permit).

## Data Availability

The datasets generated and analyzed for this study can be found in the Open Science Framework repository [https://osf.io/93x4y].
